# Occupational Therapists in Patient Navigation: A Scoping Review of the Literature

**DOI:** 10.1177/15394492231161283

**Published:** 2023-04-17

**Authors:** Kristina M. Kokorelias, Hardeep Singh, Alexandra N. Thompson, Amy E. Nesbitt, Jessica E. Shiers-Hanley, Michelle L. A. Nelson, Sander L. Hitzig

**Affiliations:** 1 St. John’s Rehab Research Program, Sunnybrook Research Institute, Sunnybrook Health Sciences Centre, Toronto, Ontario, Canada; 2 University of Toronto, Ontario, Canada; 3 Sinai Health System, Toronto, Ontario, Canada

**Keywords:** occupational therapy, scoping review, patient navigation, continuity of care, chronic illness, coordination

## Abstract

This review seeks to understand the literature on patient navigator programs (PNPs) that employ occupational therapists (OTs), including the role (conceptualization), functions (operationalization) of OTs who work as patient navigators (PNs) and the settings and populations they serve. This review also mapped the role of PNs to the 2021 Competencies for Occupational Therapists in Canada. Scoping review methodology by Arksey and O’Malley (2005) was employed. Data were analyzed thematically and numerically to identify frequent patterns. Ten articles were included. Within PNPs, OTs worked in hospitals and communities, but their role was rarely well-defined. Five competency domains (i.e., communication and collaboration, culture, equity and justice, excellence in practice, professional responsibility, and engagement with the profession) were evident in existing PNPs that included OTs. This review supports the increasing interest in OTs as PNs by demonstrating the alignment between the OT competencies and roles and functions of OTs working within PNPs.

## Introduction

Patient navigator programs (PNPs) are programs that involve a trained patient navigator (PN) to identify individual barriers to accessing care and assist with reducing delays in accessing a continuum of services to improve patient health outcomes (Wells et al., 2008). PNPs have been expanded to meet the diverse needs of care contexts (McBrien et al., 2018), yet there remains a lack of consensus regarding PN’s titles, job descriptions, credentials, competencies and training (Kelly et al., 2019). Some functions of PNs overlap with those of case managers (Kelly et al., 2019). Although the primary purpose of case managers is to fill gaps in services, such as by providing clinical consultation, the primary purpose of PNs is to help patients navigate existing health and social care services (Kelly et al., 2019). Moreover, PNs often are not restricted to a “predefined set of services,” in the way that case managers are (Dohan & Schrag, 2005).

The credentials and professional background of PNs can vary (Kelly et al., 2019). There is abundant data on the role of lay navigators (i.e., peers with lived experience; e.g., Budde et al., 2021). However, for the purposes of this review, we defined a PN as a health care professional that engages with patients and/or clients on an individual basis for a defined but ongoing period to increase access to components of the health care system and enhance chronic disease care (Kokorelias et al., 2021a). Various health care disciplines have taken on the PN role, including nurses, social workers, and occupational therapists (OTs; Kelly et al., 2019; McBrien et al., 2018). Much of the literature has explored the role of social workers (e.g., Browne et al., 2015) and nurses (e.g., Gilbert et al., 2011) in PN roles. Although OTs have served as PNs or in PNPs (Menendez, 2020), OTs as PNs remain understudied (Kelly et al., 2019). Consequently, there are no discipline-specific guidelines for OTs to deliver PNPs.

OTs may be uniquely suitable to contribute to PNP given their distinct professional training in rehabilitation, community re-integration, and functional assessment (Stout et al., 2019). OTs focus on how personal, environmental, and occupational factors intersect to influence one’s access to health services and recovery (Stout et al., 2019). As members of interdisciplinary rehabilitative teams, OTs support the assessment and management of functional impairment, provide strategies for self-management, and promote participation in meaningful occupation (Stout et al., 2019). These OT-specific skills may enhance PNPs, which ultimately might lead to improved health outcomes for adults living with chronic conditions who require ongoing care. Despite this potential for improvement, the evidence remains unclear as to the unique role OTs can have in PNPs and how they are currently contributing to existing PNPs; hence, research is needed on the role of OTs in PNPs to address this literature gap and inform the development of future PNPs.

The competencies that guide OT practice have been defined in various ways (Association of Canadian Occupational Therapy Regulatory Organizations [ACOTRO], 2021; Brown et al., 2007; Canadian Association of Occupational Therapists [CAOT], 2012; Chun et al., 2020). In Canada, the current set of competencies for OTs at all experience levels are outlined in the 2021 Competencies for Occupational Therapists in Canada (ACOTRO, 2021). This document outlines six domains of competencies: (a) occupational therapy expertise; (b) communication and collaboration; (c) culture, equity, and justice; (d) excellence in practice; (e) professional responsibility; and (f) engagement with the profession (ACOTRO, 2021). These six domains are described in Table 1.

**Table 1.** Overview of the Six Domains of Essential Competencies of Practice for Occupational Therapists in Canada.

Essential competency	Description
1—Occupational therapy expertise	The expertise that occupational therapists have to understand and support the occupational needs of clients by analyzing the gap between what people do and what they want or need to do to optimize their health and well-being, to identify opportunities to help them meet their goals in a co-constructed manner.
2—Communication and collaboration	Occupational therapists’ skills to build respectful and interpersonal relationships with clients, team members, and colleagues involved in the circle of care.
3—Culture, equity, and justice	Occupational therapists’ ability to recognize and respond to the diverse history, cultures, and social structures that influence health and occupation of their clients.
4—Excellence in practice	Occupational therapists’ ability to take responsibility for their own continuous learning and improvement to provide quality of care within their practice.
5—Professional responsibility	Occupational therapists’ ability to adhere to the best interest of clients by offering a safe, ethical, and effective practice.
6—Engagement with the profession	Occupational therapists’ expertise in contributing to the health and social systems and related evidence and research across their career trajectory.

Although several factors determine the roles of OTs in a practice setting, including their scope and organizational policies, practices, and models of service delivery, these competencies provide an overarching guide for OT practice. An exploration of the degree to which these competencies are present within the current functions of PNs can help determine the unique skillset OTs may bring to PNPs and opportunities where OT competencies are not being used.

This scoping review aims to advance our understanding of OTs as PNs by (a) identifying and describing the literature on PNPs that employ OTs, including the role (conceptualization) and functions (operationalization) of OTs who work as PNs, as well as the settings and populations they serve; and (b) mapping the identified functions of PNs on the 2021 Competencies for Occupational Therapists in Canada. Here within, we refer to the term “mapping” to thematically understand the extent, range, and nature of PNs as it relates to existing frameworks (i.e., the 2021 Competencies for Occupational Therapists in Canada) (Arksey & O’Malley, 2005).

## Method

### Protocol and Registration

Scoping review methodology with a qualitative thematic analysis was employed (Arksey & O’Malley, 2005). The protocol for this scoping review was registered (*https://doi.org/10.17605/OSF.IO/ZSW9D*). We followed the Preferred Reporting Items for Systematic Review and Meta-Analysis Protocols extension for scoping reviews (see Supplemental File 1).

### Positionality

This literature review began with the prior assumption that OTs could or should serve as PNs. The research team comprises Canadian researchers whose programs of research include the study of PNPs (KMK, MLAN and SLH), an information specialist (JH), and OT clinician-researchers (HS, ANT, ANM). The research team has attempted to support this position with a scoping review, drawing on the 2021 Competencies for Occupational Therapists in Canada.

### Information Sources

PsycINFO, OT Seeker, OVID MEDLINE, EMBASE, OT Critically Appraised Topics (CATs), and CINAHL, including CINAHL Plus with Full Text were searched on June 7, 2021. In part, our selective search strategy was informed by other existing reviews on PNPs (e.g., Kokorelias et al., 2021b). Given the inconsistency of the term “patient navigation” within the literature (Kelly et al., 2019), a scoping literature review was deemed appropriate to identify “key articles” that could significantly help clarify the role for OTs within the context of PNPs (Singh et al., 2021). The search strategy was peer-reviewed by a Medical Information Specialist (librarian) using the Peer Review of Electronic Search Strategies framework (McGowan et al., 2016) and all feedback was incorporated (see Supplemental File 2 for the search strategies). There were no date limits or restrictions on article types to get a broad scope of the literature. Search terms included “occupational therapy” and “patient navigation” to avoid confusion with case-management literature. The search was updated on August 8, 2022, following a guideline for updating reviews (Moher et al., 2008). A hand search was performed to screen the reference lists of the included articles as well as in Campbell Collaboration; Agency for Health Care Policy and Research; American Occupational Therapy Association (AOTA) Evidence Briefs; and Canadian Occupational Therapy Foundation Critical Research Literature Reviews using the same search terms. To locate gray literature, including dissertations, two authors (KMK and JH) conducted Google searches and explored websites of organizations (e.g., National Cancer Institute) in August 2022 using the same search terms.

### Eligibility Criteria

Inclusion and exclusion criteria were determined a priori. Articles were included if they were original research, commentaries, or letters to the editors and published in English. Articles had to focus on a PNP in any care setting for accessing a health service and explicitly include OTs in PNPs. Book reviews and conference abstracts were excluded, as were articles focused on the pediatric population. Disagreements were discussed with a third reviewer (SLH).

### Selection of Sources of Evidence

Four authors (KMK, HS, ANT and AEN) conducted Level 1 (title and abstract review) and Level 2 (full article text review) screening in duplicate for all search results after deduplication of search results.

### Data Charting Process

Two authors (KMK and JH) piloted a data abstraction with two articles. One author (KMK) abstracted data from the included publications using the final form developed in a team meeting prior to the pilot exercise. This final data abstraction form included study characteristics, type of article, description of PN roles and functions, and results. All data were abstracted in duplicate and then compared. Disagreements were resolved by a third reviewer (SLH). The abstraction form and procedures were informed by a previous review by Kelly et al. (2019) which identified nine key functions of PNs to build on existing literature. These functions included (a) advocacy, (b) care coordination, (c) case monitoring and patient needs assessment, (d) community engagement, (e) education, (f) administration and research activities, (g) psychosocial support, (h) navigation of services, and (i) reduction of barriers (Kelly et al., 2019, p. 27).

### Synthesis of Results

Interpretative analysis occurred in two steps. First, we focused on identifying and describing the literature on PNPs that employ OTs, including the roles (conceptualization) and functions (operationalization) of PNs and the settings and populations they serve. This was accomplished using a codebook thematic analysis, whereby all authors independently reviewed the abstracted data to identify frequent patterns (e.g., numerical counts of study design and locations), similarities, and differences between the PNPs (Braun & Clarke, 2022). Next, the authors met regularly to discuss the themes they identified and listed all roles and functions identified within each PNP. The nine functions by Kelly et al. (2019, p. 27) informed the coding on functions. Next, we interpreted and mapped the identified functions of PNs onto the 2021 Competencies for Occupational Therapists in Canada. The authors then looked at each article to determine whether there was written evidence of each competency within each article, based on their interpretation. The determined patterns of competency adherence were then used to discuss implications for future research, policy, and practice over a series of team meetings (Braun & Clarke, 2022). We then disseminated the results in the Consultation phase (described below). The numerical counts of study characteristics were synthesized and presented in the results below.

### Consultation

The preliminary findings of this work were presented to 14 key stakeholders (e.g., staff including three OTs, two managers, and two health care administrators) at one large urban hospital and one community care agency employing PNs during a series of three meetings (one during the planning of the research and twice during data analysis). These meetings provided an opportunity to receive feedback on the preliminary themes and identify actionable recommendations for future OT and PN practice, health care policy, and research.

## Results

**Figure 1.**
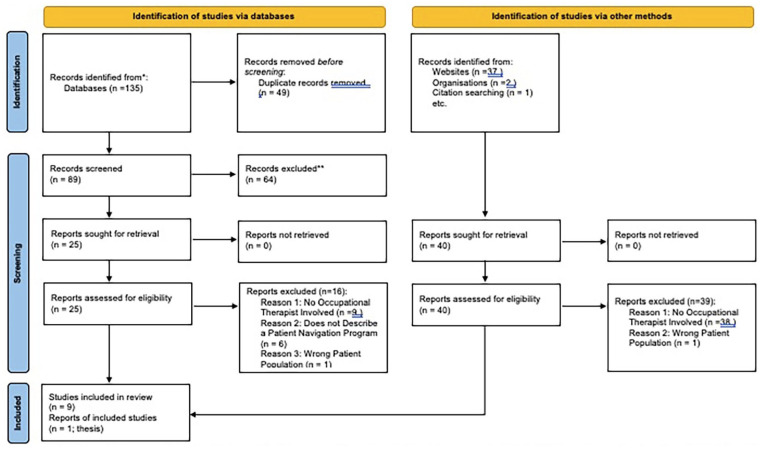
PRISMA 2020 Flow Diagram for New Systematic Reviews Which Included Searches of Databases Registers and Other Sources. *Note.* PRISMA = Preferred Reporting Items for Systematic Reviews and Meta-Analyses.

Ten articles were included in this scoping review (see Figure 1). Of the 10 included articles, one was a qualitative study, one was a mixed methods study, three were letters to the editor/commentaries (including pathway design), and five were quantitative studies. The included studies were predominantly conducted in North America (*n* = 8), with five from Canada and three from the United States. The remaining studies (*n* = 2) were conducted in the United Kingdom. All the articles were published within the last 12 years.

Almost all articles (*n* = 9) were implemented in community settings. Community settings included individuals’ homes (Milligan et al., 2018; Moore, 2013), care homes, as well as outpatient clinics (Dewan et al., 2014; Markle-Reid et al., 2020). The PNPs reported by Rosario and colleagues (2016) and Reid (2019) were implemented in both hospital and community settings. The remaining article was implemented in the hospital (Cornes et al., 2018). The characteristics of the included articles and interventions are reported in Table 2.

**Table 2.** Characteristics of Included Studies and Interventions.

Authors, country, and design	Participants and sample size (total)	Setting and primary mode of delivery	Program description	All health care providers involved in program delivery	Description of patient navigation role (e.g., program aim)
Cornes et al., 2018 United Kingdom Observational Study	Homeless people discharged from hospital *n* = 120	Hospital, Face to Face	Specialist integrated homeless hospital discharge plan	Housing workers, physicians, OTs, nurses, social workers, and peer navigators	Description Not reported (NR); peer navigators
Dewan et al., 2014 United Kingdom Evaluation	Individuals with stroke *n* = 55	Community, face to face	Joint clinics aimed at supporting stroke survivors with community rehabilitation to reduce readmissions	Stroke consultants, OTs, and stroke navigators	Hospital stroke unit and PNs worked collaboratively to improve the rate at which stroke survivors are offered interviews to aid their community re-integration
Egan et al., 2010 Canada Pretest-post-test evaluation design	Individuals with stroke and their family caregivers *n* = 73	Community, phone calls	Participants were counseled to seek treatment for the possibility of depression	OTs only	PNs synthesized available information to develop a plan of action to enhance the well-being and community integration of the individuals with stroke and family caregivers
Kwong et al., 2017 United States Letter to the editor	Older adults who identified as LGBTQ	Community, face to face	Elder LGBTQ Interprofessional Collaborative Care Program (e-clinic) was developed with an aim of improving the overall health of older LGBTQ adults by addressing their health disparities and needs	Nurse, social worker, physical therapist, OT, psychologist, and PN	PNs focused on providing health promotion and wellness, care coordination, patient navigation, and primary medical and behavioral healthcare
Markle-Reid et al., 2020 Canada Mixed-method design	Individuals with stroke *n* = 30	Community, face to face	Provision of care coordination, home visiting, and case conferences	OT, nurse, physical therapist, speech-language pathologist, social worker	PNs’ role included facilitating access to health- and social services for patients and caregivers; promoting and facilitating continuity of care; identifying and removing barriers to care; and effective and efficient use of the healthcare system
Milligan et al., 2018 Canada Qualitative	Individuals with spinal cord injury *n* = 12	Community, face to face	Comprehensive assessment of patients’ needs by the interprofessional teams who then develop a plan of care that aims to meet and fulfill the needs of participants	Nurse, exercise therapist, project manager, OT, administrative assistant, social worker, pharmacist	Met the full needs of patients including physical, social, and environmental assessments.
Moore, 2013 Canada Commentary	All citizens in a community (unspecified)	Community, phone calls	Primary Health Care & Capital District Health Authority collaboratively run community health centers to provide easy access to primary care, health promotion, health prevention, and self-management	OTs, social workers, recreation therapist	To help citizens navigate the healthcare system and the community to find and access resources to meet their health and wellness needs
Reid et al., 2019 Canada Qualitative descriptive design	Adults who identify as LGBTQ or living with mental health and addictions, cancer, diabetes, or need assistance with wellness *n* = 10	Hospital & Community, NR	Varied. PNPs were implemented in the community or hospital	Nurses, social workers, counselors, OT, a registered dietician	The PN role across all sites included advocacy, care coordination, and/or collaboration (with the family and/or care team); community engagement; administrative activities; education; providing psychosocial support; facilitating access to services and resources; and reducing barriers to care
Rosario et al., 2016 United States Evaluation Study	Individuals with spinal cord injury NR	Hospital and community, face-to-face and phone calls	PN focused on problem identification, adherence to healthy practices, and any other concerns participants had	Nurse, physician, OT, and physical therapist	The PN’s role included assisting with problem identification and resolution; monitoring adherence to healthy practices; re-integration progress, assisting with locating services in the community, and being aware of any needs that require referral/intervention
Sacco et al., 2018 United States Development of a Care Pathway	Individuals with head and neck cancer NR	Community, educational course, and an education booklet	The Care Pathway Model aims to assist with transitions in cancer care and outlined the paths of patient care experience	Physicians, nurses, speech-language pathologists, nutrition, physical therapists, OTs, social workers	To assist with care transitions by developing a clear pathway of services

*Note.* NR = not reported; LGBTQ = lesbian, gay, bisexual, transgender, or queer; OT = occupational therapists; PNP = patient navigator programs; PN = patient navigator.

The PNPs were generally focused on a specific group, medical condition, or medical program rather than on chronic illness broadly. For example, one PNP focused on people experiencing homelessness and chronic illness (Cornes et al., 2018), older adults who identified as lesbian, gay, bisexual, transgender, or queer (LGBTQ) with chronic illness (Kwong et al., 2017), individuals living with cancer (Reid, 2019; Sacco et al., 2018), mental illness/addictions (Reid, 2019) and those with spinal cord injury (Milligan et al., 2018; Rosario et al., 2016). Three PNPs targeted individuals who had a stroke (Dewan et al., 2014; Egan et al., 2010; Markle-Reid et al., 2020). One PNPs included individuals of a particular community with and without a medical condition/illness (Moore, 2013).

Nine PNPs included multi-professional navigator teams (i.e., PNs with different professional backgrounds or multiple PNs assigned to a patient) but primarily consisted of social workers as PNs. One study only included OT-trained PNs (i.e., included no other professionals as PNs) (Egan et al., 2010). One study included housing and peer navigators (Cornes et al., 2018). Most interventions were delivered in-person, and one was delivered virtually (Sacco et al., 2018).

### PNs’ Roles and Functions

Most articles (*n* = 8) described the PN role as a single-person role, although the exact titles for these roles varied (e.g., System Navigator (Markle-Reid et al., 2020), peer navigators (Cornes et al., 2018)). Two articles described the PN-role as a team-role and the titles for these roles also varied (i.e., Navigation Team Member Rosario et al., 2016) and Mobility Clinic Team Member (Milligan et al., 2018)). None of the articles provided an explicit definition of the PN role.

Functions of PNs included (a) advocacy (*n* = 3), (b) care coordination (*n* = 6), (c) case monitoring and patient needs assessment (*n* = 5), (d) community engagement (*n* = 2), (e) administration (*n* = one), (f) providing psychosocial support (*n* = 3), (g) navigation of services (*n* = 7), and (h) reduction of barriers (*n* = 5). These functions are further described in Table 3.

**Table 3:** Patient Navigators’ Functions.

Function	Description
Advocacy	Advocate on behalf of patients (Egan et al., 2010; Kwong et al., 2017; Reid, 2019).
Care coordination	Assist with care transitions (Markle-Reid et al., 2020; Sacco et al., 2018).
	Coordinate with patients, families/caregivers, and/or care team (Egan et al., 2010; Kwong et al., 2017; Markle-Reid et al., 2020; Moore, 2013; Reid, 2019).
	Provide coaching (Egan et al., 2010).
Case monitoring and patient needs assessment	Provide case management (Kwong et al., 2017).
	Assess patient needs (Dewan et al., 2014; Egan et al., 2010; Kwong et al., 2017).
	Perform ongoing assessments (Egan et al., 2010).
	Create a plan of action (Egan et al., 2010; Sacco et al., 2018).
	Monitor patients’ adherence to healthy practices (Rosario et al., 2016).
Community engagement	Collaborate with community organizations (Moore, 2013; Reid, 2019).
	Deliver outreach presentations (Reid, 2019).
Education	Provide patients with education around the healthcare system (Dewan et al., 2014; Reid, 2019; Sacco et al., 2018).
	Provide patients with education around their illness(es) & treatment(s) (Egan et al., 2010; Dewan et al., 2014; Reid, 2019; Sacco et al., 2018).
	Provide patients with education around health promotion &/or self-management (Dewan et al., 2014; Kwong et al., 2017; Moore, 2013).
	Provide family/caregiver with education (Egan et al., 2010).
	Provide the care team with education (Reid, 2019).
Administration activities	Assist with administrative activities (Reid, 2019).
Psychosocial support	Provide patients with social support (Reid, 2019).
	Provide accompaniment (Egan et al., 2010)
	Provide patients with emotional support (Egan et al., 2010; Moore, 2013; Reid, 2019).
	Counsel patients to seek mental health treatment (Egan et al., 2010).
	Provide family/caregiver with emotional support (Egan et al., 2010).
Navigation of services	Assist with referrals (Reid, 2019; Rosario et al., 2016).
	Connect patients/families/caregivers to relevant services (Egan et al., 2010; Kwong et al., 2017; Markle-Reid et al., 2020; Milligan et al., 2018; Moore, 2013; Rosario et al., 2016).
Reduction of barriers	Identify and remove barriers to care (Markle-Reid et al., 2020; Moore, 2013; Reid, 2019).
	Assist patients with problem-solving (Egan et al., 2010; Rosario et al., 2016).
	Facilitate medical transportation (Reid, 2019).
	Assist patients with complex applications (Reid, 2019).

### OT Competencies

Although none of the studies referenced any guidelines for competencies, our analysis suggested that the identified PN functions mapped onto five of the six OT competencies reported in the 2021 Competencies for Occupational Therapists in Canada were illustrated within the descriptions of PN roles. A synthesis of the identified competencies is reported in Table 4.

1. **Occupational Therapy Expertise:** None of the articles aligned with this competency, as the PN functions were never described using occupational terminology or concepts.
2. **Communication and Collaboration:** The articles described PN functions that aligned with this compotency as PNs educated clients and caregivers about the health care system as well as the clients’ illness/condition and treatment plans (Dewan et al., 2014; Egan et al., 2010; Reid, 2019; Sacco et al., 2018). Moreover, PNs collaborated with other professionals through referrals to other providers when the clients required care that fell outside their scope of practice setting (Egan et al., 2010; Kwong et al., 2017; Markle-Reid et al., 2020; Milligan et al., 2018; Moore, 2013; Rosario et al., 2016). PNs often worked closely with other providers across settings and at different organizations, including other rehabilitative providers, to ensure clients’ care needs were being met and they had access to the appropriate services (Cornes et al., 2018; Egan et al., 2010; Moore, 2013). PNs ensured their communication with others (e.g., other providers, patients, and caregivers) was effective, client-centered, and timely to help facilitate treatment plans (Milligan et al., 2018). In collaboration with other team members, PNs developed comprehensive client plans to support meaningful participation (Kwong et al., 2017; Moore, 2013). For example, PNs educated and liaised with other providers within the same setting to manage the multiple medical needs of the client (Kwong et al., 2017; Moore, 2013; Reid, 2019).
3. **Culture, Equity, and Justice:** Aligning with this competency, PNs had to advocate for their clients’ needs and equitable access to appropriate services (Egan et al., 2010; Reid, 2019; Sacco et al., 2018). Moreover, PNs helped adults who lived at home, and those with complex medical conditions navigate systematic barriers to accessing care, such as challenges with access due to mobility concerns (Cornes et al., 2018; Egan et al., 2010; Kwong et al., 2017). For example, within one PNP, navigators provided linkages to services that were aware of the sensitive needs of the LGBTQIA+ community (Kwong et al., 2017). Moreover, PNs helped connect adults to harm-reduction and recovery programs (Cornes et al., 2018).
4. **Excellence in Practice:** There was alignment with this competency as PNs were required to have practice knowledge of the health and social system as well as general health and illness (Egan et al., 2010). PNs were required to have in-depth knowledge about their practice area, including the services that clients were eligible to receive, to make appropriate referrals and assist with navigating services to support their (occupational) goals (Egan et al., 2010; Kwong et al., 2017; Markle-Reid et al., 2020; Milligan et al., 2018; Moore, 2013; Rosario et al., 2016). This knowledge was essential to supporting patients through the complexities of the health care system and for referring clients to appropriate resources to support care continuity (Egan et al., 2010; Reid, 2019). Some programs required the PN to have expertise within a specific condition (e.g., spinal cord injury) and/or designations (e.g., authorization as an Assistive Device Program Provider) (Dewan et al., 2014; Milligan et al., 2018; Moore, 2013).
5. **Professional Responsibilities:** Aligning with this competency, PNs had to respect clients’ occupational rights and choices in developing their plans (Cornes et al., 2018). PNs had to think critically while making decisions grounded in their policies, regulations, professional judgment, and clinical reasoning. Although some skills of the occupational therapy PN overlapped with other providers (e.g., social workers), OTs’ unique focus on maximizing function and occupation using multiple sources of information to create a holistic occupational profile and analysis of a client’s occupational performance were valued.
6. **Engagement with the Profession:** The articles also aligned with this competency, since—by nature of the PNPs being innovative—the included functions demonstrated commitment to quality improvement. Although many care professions equally demonstrated the same values of innovation, this suggests that there could be sharing of tasks in cases of human resource struggles (e.g., social work staffing crises whereby some social work activities, such as psychosocial assessments, can be carried out by OTs) (Milligan et al., 2018).

**Table 4.** Examples of the OT Competences in Practice Within a PNP by a PN.

Essential competencies	Examples of the OT competencies in practice within a PNP by a PN
1—Occupational therapy expertise *None of the articles explicitly mention occupational therapy	• Using knowledge within their scope of practice to assess and inform care plans (Markle-Reid et al., 2020) **Possible OT expertise inferred based on PN functions described within articles:** Maximizing function and occupation using multiple sources of information to create a holistic occupational profile and analysis of a client’s occupational performance were valued
2—Communication and collaboration	• Conducting collaborative assessments with team members (Milligan et al., 2018)
3—Culture, equity, and justice	• Advocating for clients to have access to services (Egan et al., 2010; Sacco et al., 2018)
4—Excellence in practice	• Enable clients to engage in meaningful activities (Moore, 2013)
5—Professional responsibility	• Knowledge of clinical pathway(s) that may influence participation (Dewan et al., 2014; Egan et al., 2010) • Make referrals to support care plans throughout the health care system, including what services are available to clients (Egan et al., 2010; Kwong et al., 2017; Markle-Reid et al., 2020; Milligan et al., 2018; Moore, 2013; Rosario et al., 2016)
6—Engagement with the profession	• Contributing to innovative health care delivery models, such as PNPs *(all studies)*

*Note.* OT = occupational therapists; PNP = patient navigator programs; PN = patient navigator.

## Discussion

This scoping review aimed to (a) identify and describe the literature on PNPs that employ OTs, including the roles and functions of PNs as well as the settings and populations they serve; and (b) map the identified functions of PNs onto the 2021 Competencies for Occupational Therapists in Canada. This scoping review found that only 10 articles on PNPs that explicitly include OTs. As suggested by this low number of included articles, the gap in research exploring OTs as PNs is critical to address. This review also highlighted an important gap in current PNPs, whereby no navigator roles aligned with the Occupational Therapy Expertise competency. OTs can play a valuable role in advancing PNPs given their in-depth knowledge of the health care system, their client-centered approach, as well as their unique expertise in occupation. This review highlighted the five other competencies that OTs can bring to PNPs that can supplement the role of other disciplines (i.e., communication and collaboration, culture, equity and justice, excellence in practice, professional responsibility and engagement with the profession). For example, OTs have unique competencies related to community re-integration by supporting meaningful participation through referrals to other services that can lead to improved health outcomes.

Scholars encourage the increased presence of OTs within health service reforms and novel models of care (Moyers & Metzler, 2014). The articles included in this review primarily focused on the role of social workers and not specifically the Occupational Therapy Expertise competency domain, suggesting OTs’ clinical scope is not being fully explored within the context of patient navigation research. As OTs often work in numerous professions and health care teams in a variety of settings, PNPs may provide a critical opportunity for involving occupational therapy directly into strategies for reducing fragmentation in health care (Cason, 2015). The 10 included articles demonstrated the alignment of OTs (ACOTRO, 2021) to PN roles, including the unique skillsets they bring. Specifically, this review also suggests that OTs are already well-trained to meet the needs of PN, even if current PNPs are not focusing on their occupational therapy expertise.

Occupational therapy models and theories, such as the Person-Environment-Occupation Model (PEO; Law et al., 1996) and the Canadian Model of Occupational Performance and Engagement (CMOP-E; Davis, 2017) can provide OTs as PNs with a framework to inform their approaches when helping patients overcome barriers to accessing timely services. In using these frameworks, PNs would be encouraged to consider cultural, institutional, socioeconomic, physical, and social factors affecting one’s ability to navigate health and social care services (Law et al., 1996). Moreover, the frameworks would help OTs consider all the demands placed on individuals as they take on the occupation of being a patient, such that OTs as PNs can intervene in context and increase occupational performance in the patient role (Townsend & Polatajko, 2007). These models have been shown to guide effective clinical reasoning in similar areas of practice to enable occupational participation among adults with chronic health conditions (e.g., Mjøsund et al., 2021).

Given their focus on occupational roles and preservation of engagement in meaningful activities, OTs are uniquely qualified to provide caregivers with timely education and support to enable their navigational role. Furthermore, by considering navigational roles through an occupational therapy informed, person, environment, and occupational lens, OTs can provide a holistic view of barriers to independently accessing care that may help support clients after the PNP. However, the literature suggests that OTs’ professional identity may not be well understood by other care providers, resulting in OTs being underutilized within health care models (Turner & Knight, 2015). This may be one potential explanation for the limited inclusion of OTs in PNPs, and the lack of clarity on how the role of OT-PNs is conceptualized and operationalized. Establishing a unique discourse among fellow OTs around the key competencies OTs have within the context of patient navigation can foster the development of communities of practice (Turner & Knight, 2015) and advance OTs’ professional roles within this emerging field. Further education that provides specific guidance for teaching OTs about the intricacies of patient navigation may be warranted. Research is also needed on the outcomes of occupational therapy-led PNPs from those of other professionals in clinical quality improvement initiatives.

Additional research with OTs is needed to expand their role within new practice areas, such as PNPs (Rostek, 2020). The demographics of included PNs (e.g., age, sex, employment status, employment experiences) were not reported and should be captured more fully so that differences in experience, sex, and gender could be better understood. Further information about the types of community and hospital units should be reported to better understand the contexts that help support the employment of OTs as PNs. Future research is also encouraged to explore strategies to address the key concerns of OTs in acting as PNs, including strategies to avoid the duplication of function with social workers.

As PNPs originated in the context of cancer care (Freeman & Rodriguez, 2011), it is likely that social work professionals have traditionally been selected for these roles given their strong focus on promoting social equity through social determinants of health focuses (Craig et al., 2013). Nonetheless, OTs can assist individuals in developing better problem-solving skills and civic engagement (Turcotte et al., 2020). To capture a more fulsome understanding of the role and function of OTs as PNs, further work is needed from the perspective of providers, educators, managers, patients, and decision-makers. For example, an understanding from the perspective of PNP managers as to the hiring decisions that may exclude or facilitate the participation of OTs’ need.

Overall, our findings suggest that Canada leads research on PNPs that employ OTs in PNPs. This suggests an opportunity for the occupational therapy profession in Canada to further explore their role in PNPs through continued participation by OTs. This could include participating in program evaluations or research articles on PNPs to demonstrate their effectiveness as PNs. Future research should consider organizational-level factors that affect who performs PN roles, including readiness to implement PNPs with various professionals, such that it impacts the feasibility of participation (Kokorelias et al., 2021d) and factors that influence the characteristics of ideal navigators for various patient populations (Kokorelias et al., 2021c). To achieve this aim, we urge the profession to advocate for greater OT involvement in new and established PNPs. OTs advocating for their involvement within PNPs may help contribute to their engagement with the profession. The results of this review might be used by OTs to help them demonstrate their qualifications for PN role.

It is possible that this scoping review missed relevant articles, as only English publications were included. Our review was informed by the 2021 Competencies for Occupational Therapists in Canada, which were designed to inform practice in Canada. Although the competencies overlap with others (American Occupational Therapy Association [AOTA], 2021), we acknowledge that the practices of OTs may differ by geographic location. Our search was also limited to PNPs offered in the context of chronic illness and thus may have excluded programs focusing on acute conditions that involve OTs or within contexts in which the focus on chronic illness was unclear. As PN roles are often identified using various terms, our search strategy may have excluded articles that used different synonyms, given our lack of a standard classification.

## Conclusion

This review revealed that OTs have unique competencies (e.g., the ability to recognize and respond to the diverse history, cultures, and social structures that influence health and occupation of their clients) that can advance patient navigation and evaluate the role of occupational therapy-led PNPs as a research priority. Advancing research in this area will be highly relevant in the face of ongoing barriers to health services (e.g., system fragmentation) in the years to come.

## Supplemental Material

sj-docx-2-otj-10.1177_15394492231161283 – Supplemental material for Occupational Therapists in Patient Navigation: A Scoping Review of the Literature Click here for additional data file. Supplemental material, sj-docx-2-otj-10.1177_15394492231161283 for Occupational Therapists in Patient Navigation: A Scoping Review of the Literature by Kristina M. Kokorelias, Hardeep Singh, Alexandra N. Thompson, Amy E. Nesbitt, Jessica E. Shiers-Hanley, Michelle L. A. Nelson and Sander L. Hitzig in OTJR: Occupation, Participation and Health

sj-pdf-1-otj-10.1177_15394492231161283 – Supplemental material for Occupational Therapists in Patient Navigation: A Scoping Review of the Literature Click here for additional data file. Supplemental material, sj-pdf-1-otj-10.1177_15394492231161283 for Occupational Therapists in Patient Navigation: A Scoping Review of the Literature by Kristina M. Kokorelias, Hardeep Singh, Alexandra N. Thompson, Amy E. Nesbitt, Jessica E. Shiers-Hanley, Michelle L. A. Nelson and Sander L. Hitzig in OTJR: Occupation, Participation and Health
