# Supplementary material for: Occupational Therapists in Patient Navigation: A Scoping Review of the Literature
Source: OTJR (Thorofare N J). 2023 Apr 17;44(1):117–27. doi: 10.1177/15394492231161283 (PMC10676038; doi:10.1177/15394492231161283)
Supplement: sj-docx-2-otj-10.1177_15394492231161283 – Supplemental material for Occupational Therapists in Patient Navigation: A Scoping Review of the Literature [file sj-docx-2-otj-10.1177_15394492231161283.docx]

**Supplemental File 2: Sample Search Strategy**

**Ovid MEDLINE(R) Epub Ahead of Print, In-Process & Other Non-Indexed Citations, Ovid MEDLINE(R) Daily and Ovid MEDLINE(R) <1946 to Present>**

| Search history sorted by search number ascending | | | |
| --- | --- | --- | --- |
| **#** | **Searches** | **Results** | **Type** |
| 1 | exp Occupational Therapy/ or occupational therap*.tw. or exp Occupational Therapists/ | 20360 | Advanced |
| 2 | patient navigat*.ti. | 505 | Advanced |
| 3 | care navigat*.ti. | 40 | Advanced |
| 4 | nav* program*.tw. | 409 | Advanced |
| 5 | navigator*.tw. | 3513 | Advanced |
| 6 | navigation.tw. | 25488 | Advanced |
| 7 | 2 or 3 or 4 or 5 or 6 | 28250 | Advanced |
| 8 | 1 and 7 | 33 | Advanced |
| 9 | limit 8 to yr=" -Current" | 33 | Advanced |

**Embase Classic+Embase**

| Search history sorted by search number ascending | | | |
| --- | --- | --- | --- |
| **#** | **Searches** | **Results** | **Type** |
| 1 | exp Occupational Therapy/ or occupational therap*.tw. or exp Occupational Therapists/ | 38168 | Advanced |
| 2 | patient navigat*.ti. | 910 | Advanced |
| 3 | care navigat*.ti. | 63 | Advanced |
| 4 | nav* program*.tw. | 907 | Advanced |
| 5 | navigator*.tw. | 6746 | Advanced |
| 6 | navigation.tw. | 36549 | Advanced |
| 7 | 2 or 3 or 4 or 5 or 6 | 41783 | Advanced |
| 8 | 1 and 7 | 56 | Advanced |
| 9 | limit 8 to yr=" -Current" | 56 | Advanced |

**APA PsycInfo**

| Search history sorted by search number ascending | | | |
| --- | --- | --- | --- |
| **#** | **Searches** | **Results** | **Type** |
| 1 | exp Occupational Therapy/ or occupational therap*.tw. or exp Occupational Therapists/ | 12709 | Advanced |
| 2 | patient navigat*.ti. | 162 | Advanced |
| 3 | care navigat*.ti. | 15 | Advanced |
| 4 | nav* program*.tw. | 165 | Advanced |
| 5 | navigator*.tw. | 1000 | Advanced |
| 6 | navigation.tw. | 9568 | Advanced |
| 7 | 2 or 3 or 4 or 5 or 6 | 10221 | Advanced |
| 8 | 1 and 7 | 19 | Advanced |
| 9 | limit 8 to yr=" -Current" | 19 | Advanced |

The string for CINAHL (12), OTSeeker (5), OT CATS (0), and Scopus (10) was as follows:

(Occupational therap*) AND (“patient nav*” OR “patient nav* program*” OR “care nav* program*” OR “care nav*” OR “nav* program*” OR navigat* OR “occupational therpa* nav*” OR “occupational therpa* nav* program*” OR “occupational therap* care nav*”)
